# Uptake of online HIV-related continuing medical education training among primary care providers in Southeast United States, 2017–2018

**DOI:** 10.1080/09540121.2020.1822986

**Published:** 2020-09-27

**Authors:** Kirk D. Henny, Christopher C. Duke, Madeline Y. Sutton

**Affiliations:** a Division of HIV/AIDS Prevention, National Centers for HIV, Viral Hepatitis, STD and TB Prevention, Centers for Disease Control and Prevention, Atlanta, GA, USA;; b Altarum Institute, Ann Arbor, MI, USA;; c Department of Obstetrics and Gynecology, Morehouse School of Medicine, Atlanta, GA, USA

## Abstract

Primary care providers play a vital role for HIV prevention and care in high burden areas of the Southeast United States. Studies reveal that only a third of these providers had previous HIV-related training. We evaluated the effects of targeted online continuing medical education training on HIV-related knowledge, attitudes and practices among providers in the Southeast. During April 2017–February 2018, we administered baseline and six-month follow-up surveys to assess changes attributed to online training among a representative sample of providers from six Southeast locations. Data were analyzed using logistic regression analysis (*p* < 0.05). Baseline and follow-up surveys were completed by 349 participants (61.2% female, 64.6% white, 69.6% physicians, and 27.5% aged 40 years or less); 18% (*n* = 63) of whom visited online training websites sent following the baseline survey. Comparing baseline versus follow-up responses, providers who completed online training were half as likely to identify “patients’ age” (30% vs. 15%) and “patients’ race” (3% vs. 1.4%) as barriers to discussing sex with clients; survey responses by participants who declined training remained unchanged. Based on baseline versus follow-up responses, providers who visited online training websites were more likely to become familiar with preexposure prophylaxis (PrEP) (38% vs. 58%); participants who declined training remained unchanged at 45%. No impact on clinical practices such as PrEP prescriptions was identified. Targeted online training can enhance HIV readiness and should be explored for providers in the Southeast, particularly for enhancing PrEP service delivery.

## Introduction

Primary care providers (PCPs) are uniquely positioned to inform, educate, and deliver HIV-related services. However, PCPs may not be prepared to provide such services (Blackstock et al., 2017); recent studies indicate that behaviors, attitudes, and practices among providers may be barriers to patient accessing needed HIV services (Puro et al., 2013; Sison et al., 2013; Tripathi et al., 2012). Online continuing medical education (CME) training provides opportunities for PCPs to foster the adoption of new skills and knowledge (Casebeer et al., 2002; Harris et al., 2010). We evaluated the effects of offering free online CME trainings that target deficits in HIV-related knowledge, attitudes, and practices among a representative sample of providers in high HIV-burden Southeast locations.

## Methods

### K-BAP Study

We conducted the Knowledge, Behaviors, Attitudes and Practices of HIV-Related Care among Providers in the Southeast (K-BAP) Study (2017–2018), an online survey of PCPs in the Southeast. Study locations were selected based on: (1) being metropolitan statistical areas (MSAs) in Southeast U.S., (2) having a large African American population (>20% of adults aged 18–54 years), (3) and having high HIV burden (HIV incidence >25 cases per 100,000 persons and prevalence >300 cases per 100,000 persons) according to 2014 HIV surveillance data (Centers for Disease Control and Prevention, 2015). Selected MSAs included Atlanta, GA; Baltimore, MD; Baton Rouge, LA; Miami, FL; New Orleans, LA; and Washington, DC. Eligible PCPs consisted of physicians, nurse practitioners and physician assistants who practiced in clinical areas that involve direct primary care to clients (Appendix 1).

### Sampling, study population, and participant recruitment

Sampling frame of the study was derived from the IQVIA® provider database, which contains a census of currently active health care providers in the U.S. (IQVIA, 2018). We acquired data for a sampling frame consisting of 36,489 providers (within the selected MSAs) in practice during January 2017. We selected a representative sample of 7330 providers for survey fielding. We stratified the survey sample by MSAs (*n*=6) and provider types (*n*=3). Providers received postal and electronic mail invitations with weblinks to online informed consent and the 56-item web survey (with unique password), followed by additional email and phone reminders. Participants who completed the baseline (BL) assessment received a $20 cash incentive via postal mail.

### Intervention and 6-month follow-up survey

Following BL web survey, participants were provided a unique web link to a recommended free online course (length: 1–3 hours; credit hours: 0.25–1.0 hours) that specifically addressed their HIV-related training deficit. The recommended course was selected from a pre-determined list of HIV-focused CME credit-eligible modules from the International Antiviral Society U.S.A. (IASUSA) available between February and December 2017 using a computer-based algorithm based on participants’ BL survey responses. These courses focused on the following topics: Antiretroviral Therapy in HIV patients, STIs in HIV patients, and HIV in hard-to-reach populations. Participants who completed the BL survey (regardless of CME training participation) were invited to complete a 6-month follow-up (6M) survey consisting of core questions from the BL survey using similar fielding schedule. The 6M survey period closed on February 28, 2018.

### Statistical analyses

Changes in results between the BL survey and 6M survey were assessed with multivariate binary logistic regression (Version 9.4; Cary, NC). The knowledge-related outcomes included familiarity with non-occupational post-exposure prophylaxis (nPEP), patient ever requested nPEP, familiarity with preexposure prophylaxis (PrEP), and patient ever requested PrEP. Attitude-related outcomes comprised of the following: barriers to discussing sexual education, sexual orientation or sexual risk with patients; and patient characteristics that make PCPs uncomfortable discussing sex with patients (gender, age, race, sexual orientation, or other). Outcomes related to clinical practices included the following: obtaining patients’ sexual risk, mental health or substance use histories; conducting depression screening; conducting screening for HIV (ever, frequency, test type), syphilis, or hepatitis C; and prescribing nPEP or PrEP. Independent predictor variables included the BL survey response for the outcome, participation in CME training following the BL survey, and the interaction of CME training with the BL survey response. The original survey questions are listed in Appendix 2.

## Results

We received 995 provider responses, of which 820 were from eligible providers and were included in the analysis. We calculated the adjusted response rate as 29.6% (AAPOR RR4: excludes known and estimated ineligible respondents from denominator) (The American Association for Public Opinion Research, 2016). A total of 349 providers completed both BL and 6M surveys; respondents were comprised of 61.2% females, 64.6% whites, 69.6% physicians, and 27.5% persons aged 40 years or less. Of these, 63 (18.1%) providers elected to participate in CME training following the BL survey, and 286 (81.9%) did not participate in CME training. All 349 providers who completed BL and 6M surveys were included in the analysis; providers who only completed the BL survey were not included.

### Overall effect CME training

**Figure 1.**
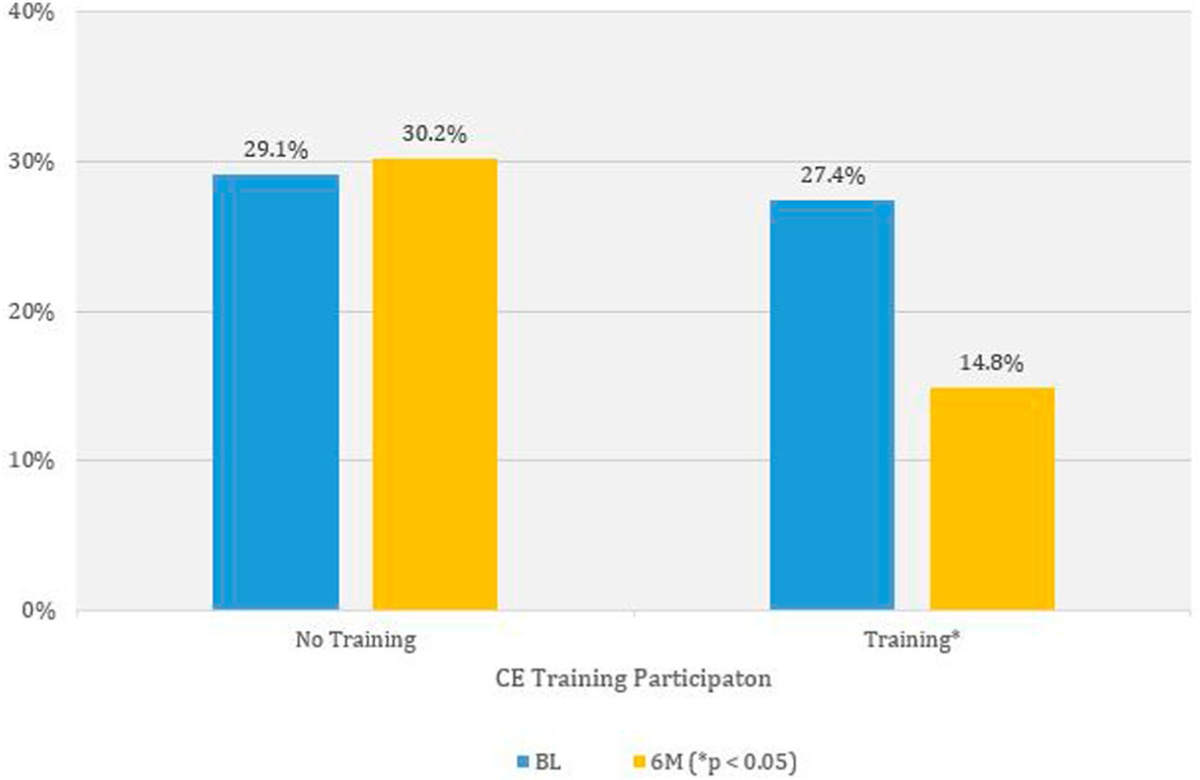
Weighted percentage of primary care providers agreeing patients age is a barrier to discussing sexual risk by survey time and consuming medical education training in selected southern states – Knowledge, Barriers, Attitudes and Practices of HIV Providers in Southeast Study, 2017–2018.

**Figure 2.**
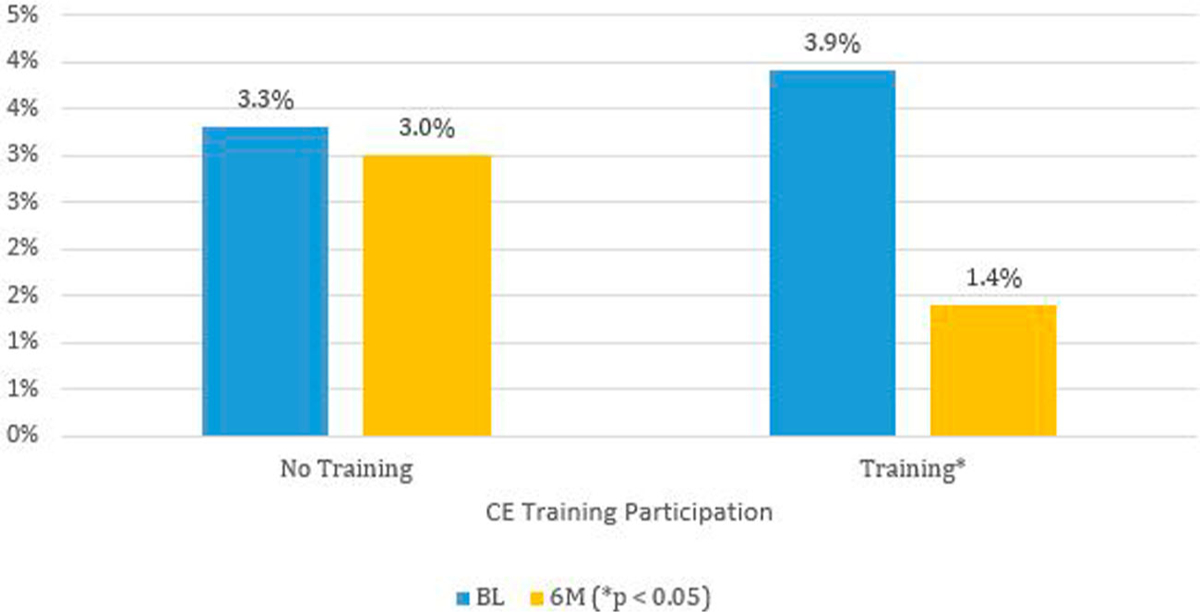
Weighted proportions of primary care providers agreeing patients race is a barrier to discussing sexual risk by survey time and consuming medical education training in selected southern states – Knowledge, Barriers, Attitudes and Practices of HIV Providers in Southeast Study, 2017–2018.

Providers who opted to initiate CME training became less likely to agree that patient age is a barrier to discussing sexual risks (*p* = 0.02), dropping from 30.2% to 14.8%, a reduction of approximately one half. The responses among those who did not initiate CME training had little difference, with 29.1% at BL and 27.4% at 6M (Figure 1). The logistic regression results are presented in Table 1. Providers who opted to initiate CME training became less likely to agree patient race is a barrier to discussing sexual risks (*p* = 0.03), dropping from 3.0% to 1.4%, a reduction of approximately one half (Figure 2). Those who did not initiate CME training were slightly more likely to agree race is a barrier, from 3.3% to 3.9% at 6M, suggesting a significant interaction between CME participation and a BL belief that race is a barrier to discussing sexual risks. The logistic regression results are presented in Table 1.

**Figure 3.**
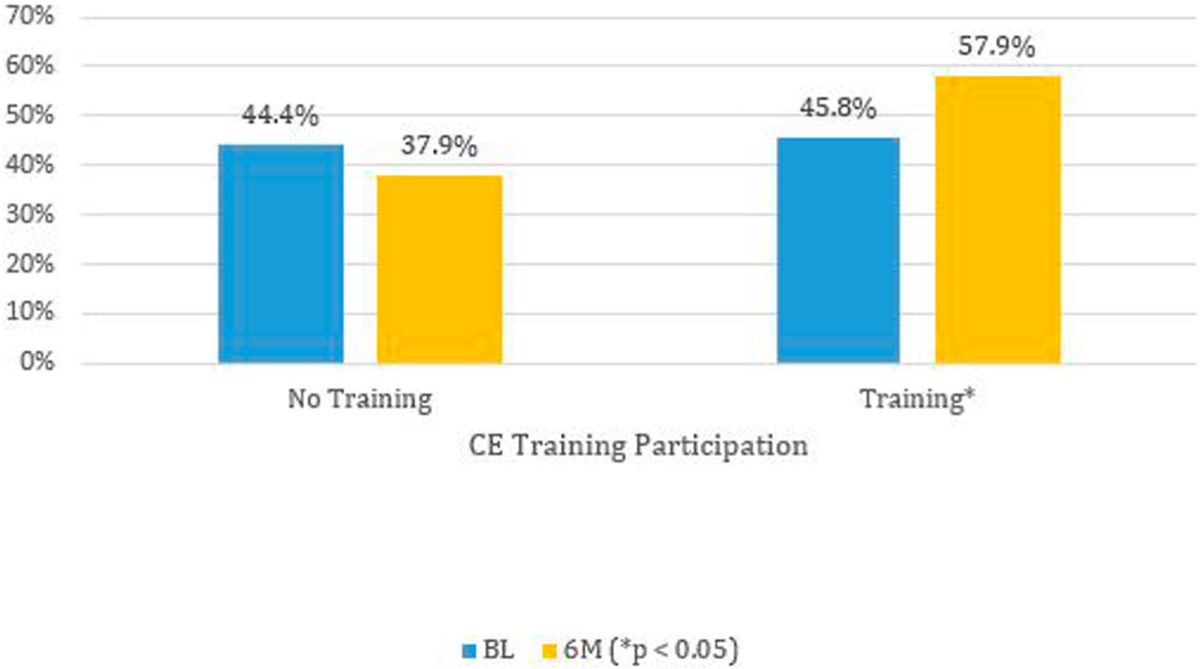
Weighted percentage of self-reported “good understanding” of PrEP by survey time and consuming medical education training in selected southern states – Knowledge, Barriers, Attitudes and Practices of HIV Providers in Southeast Study, 2017–2018.

Providers who initiated CME training became more likely to self-report having a “good understanding” of PrEP at 6M (*p* = 0.03), increasing from 37.9% to 57.9%, an increase just over 50% (Figure 3). Those who did not initiate CME training showed little change, from 44.4% to 45.8% at 6M. Logistic regression results are presented in Table 1. Taken together, these results suggest that participation in HIV CME training was associated with improvements in provider perceptions that age or race is a barrier to discussing sexual risk and greater self-reported understanding of PrEP.

## Discussion

Our analyses revealed that CME training improved only a few of the measures assessed among our sample of PCPs in selected Southeast locations. PCPs who participated in targeted CME trainings were less likely to report age and race as barriers for discussing sexual risk with their patients. This result shows the potential impact that the targeted online CME trainings can have on improving patient communication regarding HIV-related topics such as sexual health screening and ART adherence particularly among younger aged men and persons of color (Carey et al., 2018, 2019; Fisher et al., 2018; Meanley et al., 2015). Conversely, other analyses show that providers with limited HIV-stigma training were more likely to exhibit stigmatizing behaviors toward their patients (Geter et al., 2018). Given this context, the impact of targeted online CME training on improving age and race-related barriers to discussing sex risk with patients is encouraging.

We also found that PCPs who participated in targeted online CME training also improved general familiarity with PrEP; this finding is particularly salient. PrEP uptake among persons with clinical indications (Centers for Disease Control and Prevention, 2014) has been low particularly in the Southeast and among those with PCPs reporting low PrEP knowledge (Mullin et al., 2016; Pet-roll et al., 2017). Our analyses reveal that targeting CME trainings can be useful for improving self-reported provider knowledge of PrEP.

We did not find changes in HIV-related clinical practices (i.e., PrEP prescriptions) related to CME training participation. The incongruence between training and clinical practices has been noted in other studies and warrants further examination (de Munnik et al., 2017; Douglas et al., 2016). Although not examined in our analyses, other studies found that successful translation of PCP training to practice involved factors such as organization-wide capacity assessments (Kadu & Stolee, 2015; Shook et al., 2016). Also, providers may have simply taken HIV-related training elsewhere. Further investigations that explore this dynamic are warranted.

There are some limitations to note regarding analysis interpretation. The 29.6% adjusted BL response rate may be perceived as low. However, our study’s response rate was comparable to those from similar surveys among providers who had not been engaged in previous studies or projects with the study investigators (Jensen et al., 2017; McManus et al., 2014; Shirts et al., 2009; Ward et al., 2011). Also, we were unable to monitor participants’ CME training progression nor completion because the course websites were managed independently by online training providers.

## Conclusions

Online CME trainings may be effective tools for improving HIV-related knowledge and attitudes among PCPs in high HIV burden areas. Future efforts should include strategies for targeting appropriate PCPs for these trainings.

## Figures and Tables

**Table 1.** Statistically significant binary logistic regression maximum likelihood estimates of CME training impact on measures of clinical behaviors, attitudes and practices among primary care providers in selected southern states – Knowledge, Behaviors, Attitudes and Practices of HIV-Related Care among Providers in the Southeast (K-BAP) Study, 2017–2018 (*n* = 349).

Measures	Parameter	DF	Estimate	Std. Error	Wald Chi Sq	*P*
Barriers to discussing sex (Age)	CME Training	1	1.4378	0.6226	5.3326	0.0209
	BL: Age is Barrier	1	−1.5688	0.419	14.0201	0.0002
	CME Training*BL: Age is Barrier	1	−0.5598	0.643	0.7578	0.3840
Barriers to discussing sex (Race)	CME Training	1	2.3054	1.0859	4.5071	0.0338
	BL: Race is Barrier	1	14.2648	0.6081	550.3085	<.0001
	CME Training*BL: Race is Barrier	1	−19.353	1.923	101.2874	<.0001
PrEP Familiarity	CME Training	1	−1.1148	0.5214	4.5724	0.0325
	BL: PrEP Understanding	1	−2.428	0.6534	13.8077	0.0002
	CME Training*BL: PrEP Understanding	1	0.9443	0.8601	1.2055	0.2722

PrEP: pre-exposure prophylaxis; DF: degrees of freedom; CME: continuing medical education; BL: baseline survey.

## Appendices

### Appendix 1. Eligible medical specialties of primary care providers selected for the Knowledge, Behaviors, Attitudes and Practices of HIV-Related Care among Providers in the Southeast (K-BAP) Study, 2017–2018.

1. Adolescent Medicine
2. Community Health
3. Emergency Medicine Specialist
4. Family Practitioner
5. Women’s Health Specialist
6. Geriatrician
7. General Practitioner
8. Gynecologist
9. Infectious Disease Specialist
10. Internal Medicine
11. Internal Medicine/Pediatrics
12. Obstetrics & Gynecology
13. Obstetrics
14. Pediatrician
15. Primary Care
16. Preventive Medicine Specialist

### Appendix 2. Selected survey questions used for analysis–knowledge, behaviors, Attitudes and practices of HIV-Related care among providers in the Southeast (K-BAP) Study, 2017–2018.

#### Patient sex history and risk

Do you obtain a sexual history and risk assessment from your patients? Please select the option that best characterizes your approach.

- I routinely obtain a sexual history at the first encounter and update it on a regular (e.g., annual) basis.
- I routinely obtain a sexual history at the first encounter and update if new information is obtained.
- I obtain an initial sexual history as needed and update it if new information is obtained.
- I document a sexual history only when volunteered by the patient.
- I do not document sexual histories.
- Other, Specify (—)

For what proportion of patients you see for continuous or repeated care do you perform the following?

**Table:**

	Most or all	About half	Few or none
Ask about number and gender of sexual partners?			
Ask about frequency and types (vaginal, anal, oral) of sex?			
Explore opportunities for safer sex counseling at each visit for sexually active patients?			

#### Barriers to patient-provider sex-related Discussions

Please indicate the extent to which you agree that the following issues pose a barrier to discussing sexual education, sexual orientation, or sexual risks with your patients?

**Table:**

	Strongly agree	Somewhat agree	Neither agree nor disagree	Somewhat disagree	Strongly disagree
I do not have enough time.					
I am not reimbursed for my time.					
My patients will not feel comfortable discussing sex.					
I do not feel comfortable discussing sex with some patients.					
Not relevant to reason for visit.					

[If agree about being uncomfortable discussing sex with some patients] Which of these patient characteristics make you uncomfortable discussing sex with patients? Please select all that apply.

- Gender
- Age
- Race
- Sexual orientation
- Other, Specify (—)

#### Patient mental health and substance use history

Do you obtain a mental health history from your patients? Please select the option that best characterizes your approach.

- I routinely obtain a mental health history at the first encounter and update it on a regular (e.g., annual) basis.
- I routinely obtain a mental health history at the first encounter and update it if new information is obtained.
- I obtain an initial mental health history as needed and update it if new information is obtained.
- I document a mental health history only when volunteered by the patient.
- I do not document mental health histories.
- Other, Specify (—)

Do you obtain a substance (drug and alcohol) use history from your patients? Please select the option that best characterizes your approach.

- I routinely obtain a substance use history at the first encounter and update it on a regular (e.g., annual) basis.
- I routinely obtain a substance use history at the first encounter and update it if new information is obtained.
- I obtain an initial substance use history as needed and update it if new information is obtained.
- I document a substance use history only when volunteered by the patient.
- I do not document substance use histories.
- Other, Specify (—)

Which of the following best describes your practice on depression screening:

- I routinely conduct depression screening on all patients.
- I conduct depression screening only if the patient has a personal history or family history of depression
- I conduct depression screening only if the patient has signs or symptoms suggestive of depression.
- I conduct depression screening if the patient has a personal history or family history of depression or if the patient has signs or symptoms suggestive of depression.
- I conduct depression screening in situations other than as described in choice B, C, or, D above. (please specify in what situations you would screen for depression)

#### HIV/STI screening practices

Do you offer HIV testing to your patients?

- Yes (SKIP to 18)
- No

How do you offer tests for HIV? Please select the response that best characterizes your practice.

- Repeated testing (3–12 months) based on patient behavior (e.g., new sexual partners, sex without condoms outside a monogamous relationship, multiple sexual partners,)
- Routine, opt-out (You tell all patients 15–65 years old that you will be performing an HIV test; they may refuse)
- Risk-based or targeted, opt-out (If you feel the patient is at risk for acquiring HIV, you tell the patient that you will be performing an HIV test; they may refuse)
- Risk-based or targeted, opt-in (If you feel the patient is at risk for acquiring HIV, you ask the patient if would like an HIV test; they must accept)
- Routine, opt-in (You ask all patients 15–65 years old if they would like an HIV test; they must accept)
- Patient initiated (HIV testing is provided to any patients who request HIV testing)
- Other, Specify (—)

How often do you offer HIV testing to the following patients?

**Table:**

	Each clinical visit	More than once per year, but not every visit	Annually	Once, documented in medical record	Never, I do not conduct clinical testing, but I refer to others	Never, I do not conduct clinical testing or refer to others
Patients who are sexually active with more than one partner						
Men who have sex with other men						
Patients who identify as transgender						
Patients who use injection drugs						
Patients who have been diagnosed with an STD						
Patients with signs and symptoms of an STD						

Do you offer rapid HIV testing, either oral swab or blood (e.g., OraQuick and Uni-Gold) in your practice?

- Yes, it is my first-line test for all patients receiving HIV testing
- Yes, I use this for many of my patients receiving HIV testing
- Yes, but rarely
- Never

Do you offer routine HIV testing through standard venipuncture sent to a lab?

- Yes, it is my first-line test for all patients receiving HIV testing
- Yes, I use this for many of my patients receiving HIV testing
- Yes, but rarely
- Never

Have you ever ordered testing which specifically tests for new HIV infection after a recent exposure?

- Yes
- No

When a patient presents with signs and symptoms compatible with any sexually transmitted disease or a report of an STD in a sex partner, do you include a test for syphilis?

- Yes, routinely, before STD diagnosis is confirmed
- Yes, routinely, only after STD diagnosis is confirmed
- Yes, occasionally
- Rarely or Never

Do you routinely screen for hepatitis C among your patients living with HIV infection?

- Yes
- No

#### Familiarity and prescribing nPEP and PrEP

Are you familiar with the concept of providing post-exposure prophylaxis (PEP) for sexual exposure to HIV?

- I have a good understanding of the concept.
- I have heard about the concept but know little about it.
- I have never heard about the concept.

Has a patient ever requested post-exposure prophylaxis (PEP) for sexual exposure?

- Yes
- No
- I do not remember

Have you ever prescribed post-exposure prophylaxis (PEP) for sexual exposure?

- Yes,
- No
- I do not remember

How familiar are you with the concept of pre-exposure prophylaxis (PrEP) in order to prevent HIV infection?

- I have a good understanding of the concept.
- I have heard about the concept but know little about it.
- I have never heard about the concept.

Has a patient ever requested pre-exposure prophylaxis (PrEP)?

- Yes
- No
- I do not remember

Have you ever prescribed any form of pre-exposure prophylaxis (PrEP) to a patient?

- Yes
- No
- I do not remember

#### Other measures

Do you provide condoms to the patients in your practice?

- No
- Yes, by request
- Yes, openly available
- Yes, patients are encouraged to take condoms
- I’m not certain if condoms are available

Do you provide primary care for your HIV-infected patients (i.e., Point of first contact, comprehensive care, and emphasis on prevention and coordination of care)?

- Yes
- No (SKIP to 38)

Do you provide HIV care in partnership with an Infectious Disease doctor?

- Yes
- No

When you diagnose someone with HIV or an STD, how do you (or your practice) handle partner notification (informing sex partners of my patient of a possible recent HIV exposure)?

- I (or my staff) make calls to partners
- I (or my staff) notify the Department of Health for assistance with partner notification
- The Department of Health will automatically handle partner notification
- I encourage my patient to notify their partners
- Other, Specify (—)
- My practice does not perform partner notification
